# A bibliometric analysis of the top 100 most cited articles on pineal parenchymal tumors worldwide

**DOI:** 10.1016/j.bas.2025.105608

**Published:** 2025-09-23

**Authors:** Alaa Samkari, Abdulaziz Hamzah, Badr Hafiz, Fayez Alshehri, Anfal Aman Felimban, Tarfah Bassam Albedaiwi, Obada Ahmad Banjar, Abdullah Bandar Alsharif, Hani Mahboob

**Affiliations:** a Department of Medicine, Faculty of Medicine, King Saud Bin Abdulaziz University for Health Sciences, Jeddah, Saudi Arabia; b Department of Pathology and Laboratory Medicine, Faculty of Medicine, Ministry of National Guard-Health Affairs, Jeddah, Saudi Arabia; c King Abdullah International Medical Research Center, Jeddah, Saudi Arabia; d Department of Neurosciences, Section of Neurosurgery, King Faisal Specialist Hospital and Research Centre, Jeddah, Saudi Arabia; e Department of Neurosciences, Section of Neurosurgery, Ministry of the National Guard-Health Affairs, Jeddah, Saudi Arabia; f College of Medicine, King Saud Bin Abdulaziz University for Health Sciences, Jeddah, Saudi Arabia; g Department of Neurosurgery, King Fahad General Hospital, Ministry of Health, Jeddah, Saudi Arabia

**Keywords:** Pineal parenchymal tumor, Pineocytoma, Pineoblastoma, Bibliometric analysis, Bibliometric study, Citation analysis, Desmoplastic myxoid tumor

## Abstract

**Background:**

Pineal parenchymal tumors are rare central nervous system neoplasms, accounting for less than 1 % of all CNS tumors. We conducted a bibliometric analysis of the 100 most cited articles on pineal parenchymal tumors to identify research trends and highlight influential contributions. Tumor types analyzed included pineocytoma, pineal parenchymal tumor of intermediate differentiation (PPTID), pineoblastoma, and papillary tumor of the pineal region (PTPR), along with the recently classified desmoplastic myxoid tumor, SMARCB1-mutant. To our knowledge, this is the first bibliometric analysis dedicated to pineal parenchymal tumors.

**Methodology:**

Articles were identified through the Web of Science database without time restriction, screened by title, keyword, and abstract. Tumor categorization followed the WHO 5th edition for CNS tumors. Articles discussing non-parenchymal pineal region tumors were excluded. Citation count determined article selection. Data collected included publication year, authorship, journal source, study category, pathology focus, and article type. Data collection and analysis was performed using Microsoft Excel and Google Sheets.

**Results:**

A total of 573 articles were retrieved; the top 100 were analyzed, totaling 3845 citations. These articles were published in 42 journals from 16 countries and 74 institutions. The oldest article was published in 1970 and the most recent in 2021. Acta Neuropathologica contributed the most publications (14). The United States accounted for the highest number of articles (34) and citations (1,326). Histopathology was the most studied category (36 %), and case reports and series were the predominant article types. The papillary tumor of the pineal region was the most frequently discussed pathology.

**Conclusion:**

This bibliometric analysis reveals a dominance of histopathological studies and reveals critical gaps in clinical studies, surgical management, and patient outcomes. Targeted future research in these areas is needed to improve diagnosis, management, and patient care.

## Highlights

• A bibliometric analysis of the top 100 most cited articles on pineal parenchymal tumors.
• Histopathology was the predominant research focus among the top-cited articles.
• Most articles were published between 2000 and 2010, mainly from the United States.
• Significant gaps were identified in clinical studies, surgical management, and outcomes.
• Future research should focus on treatment strategies and clinical outcome reporting.

## 1 Introduction

The primary role of the pineal gland is to perceive environmental stimuli related to the light-dark cycle, leading to the secretion of the melatonin hormone. Melatonin production reflects the internal circadian rhythm and is typically released during the dark phase of the daily cycle (Arendt et al., 2000).

Pineal parenchymal tumors are a rare category of central nervous system (CNS) neoplasms, characterized by diverse histologic features and clinical presentations. These tumors comprise less than 1 % of all CNS tumors and encompass a spectrum of entities, ranging from World Health Organization (WHO) Grade 1 Pineocytomas to WHO Grade 2–3 pineal parenchymal tumors of intermediate differentiation (PPTIDs), and Grade 4 Pineoblastomas, with a primary predilection for pediatric patients (Liu et al., 2021). Notably, the WHO CNS fifth edition classification has been extended to include desmoplastic myxoid tumor of the pineal region, SMARCB1-mutant**,** which represents a rare tumor lacking histopathological indications of malignancy and is characterized by SMARCB1 mutation (David N Louis and others, 2021).

Bibliometric analyses have gained increasing recognition as invaluable tools for assessing scholarly impact, identifying publication trends, and addressing knowledge gaps in specific fields, thereby guiding future research endeavors. While several bibliometric analyses have been conducted on various neurosurgery topics, including vascular, spinal, oncological, and trauma-related subjects, to the best of our knowledge, no bibliometric analysis focusing on pineal parenchymal tumors had been published before (Hamzah et al., 2023). Consequently, in this study, we present a bibliometric analysis of the top 100 cited articles on pineal parenchymal tumors.

## 2 Methodology

### 2.1 Search strategy

The study's analysis adhered to PRISMA guidelines, and papers about different kinds of pineal parenchymal tumors were screened using the Web of Science (WoS) search engine. The search strategy involved specific titles, keywords, and abstracts, employing terms such as Pineal Parenchymal Tumor, Pineocytoma, Pineoblastoma, Papillary tumor of the pineal region, Desmoplastic myxoid tumor of the pineal region, and SMARCB1-mutant, without time restriction.

### 2.2 Classification system

The classification of the articles into distinct pineal parenchymal tumor subtypes was based on the fifth edition of the WHO Classification of Central Nervous System Tumors published in 2021.

### 2.3 Screening and eligibility

There were 573 articles found in the search. Following duplicate removal and preliminary screening by abstract and title, full-text reviews of the articles were conducted to determine eligibility. Clinical studies that specifically addressed pineal parenchymal tumors, case reports/series, reviews, and original articles met the inclusion criteria. The following were among the exclusion criteria: articles that focus mostly on pineal tumors that are not parenchymal, veterinary research, Articles or abstracts from conferences are not accessible online.

### 2.4 Data extraction and analysis

The search results were sorted by citation count in descending order. The authors followed a thorough selection process, including title and abstract screening, followed by full-text review to identify the most cited articles that met the criteria. Two independent authors (Obada Banjar and Abdullah Alsharif) performed title screening to include relevant articles that fit the predefined inclusion criteria and exclude those that did not align with them. Another set of two independent authors (Anfal Felimban and Tarfah Albedaiwi) obtained the full-text of selected articles and collected data on publication year, authors, journal, category (histopathology, review, management, etc.), pathology studied, and article type (case report/series, randomized controlled trial, systematic review, review, retrospective, etc.).

Inclusion criteria included articles discussing any type of pineal parenchymal tumors, while exclusion criteria excluded articles predominantly discussing topics other than pineal parenchymal tumors, veterinary studies, articles not available online, and conference abstracts. When available, articles with only abstracts were reviewed to assess relevance; however, none were deemed sufficiently relevant for inclusion.

Data collection and analysis were conducted using Microsoft Excel and Google Sheets.

## 3 Results

### 3.1 Search results

**Fig. 1:**
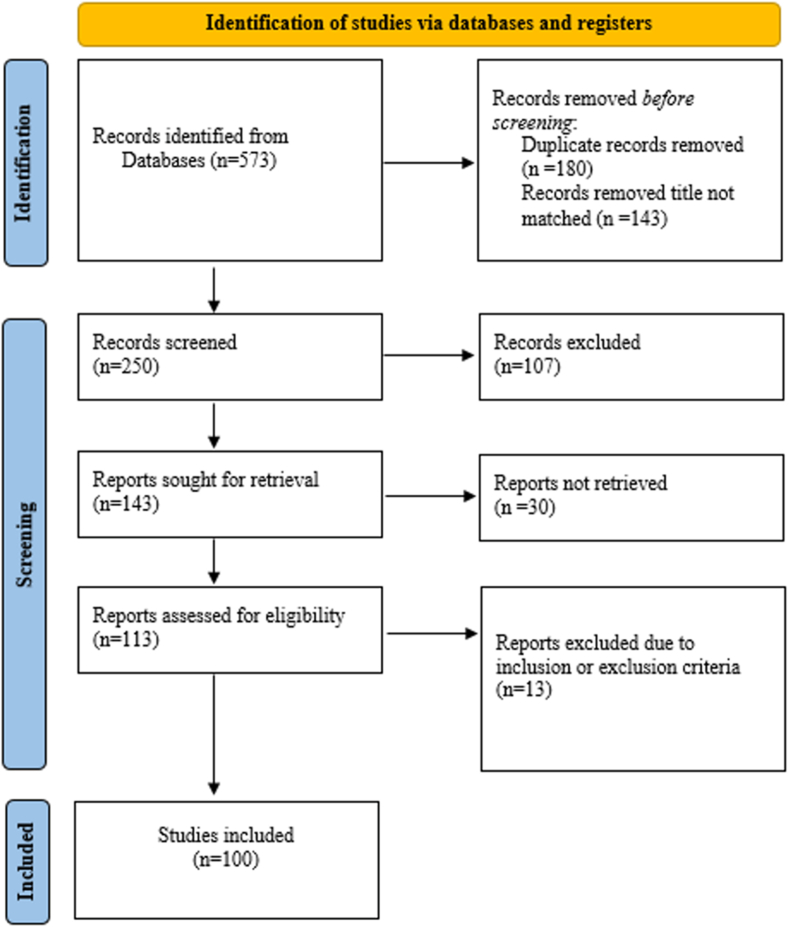
PRISMA flow diagram.

The Web of Science (WoS) search yielded 573 articles, and the top one hundred most cited ones were extracted and analyzed (Fig. 1). The top 100 most cited articles in pineal parenchymal tumors totaled 3845 citations, averaging 38.45 citations, and ranging from 17 to 152 citations per article. The articles were published in 42 different journals, from 16 countries, and 74 institutions. The average citation per year (C.Y) was 2.7, ranging from 0.4 to 12. The oldest article was published in 1970, with the most recent one published in 2021 (Table 1).

**Table 1:** Top 100 cited articles of Pineal Parenchymal Tumors.

Publication Year	Authors	Article Title	Journal	CC	C.Y
**1979**	Herrick, Mk; et al.	Cytological Differentiating Potential Of Pineal Parenchymal Neoplasms (True Pinealomas) - Clinico-Pathological Study Of 28 Tumors	Brain: A Journal Of Neurology	152	3.5
**1993**	Schild, Se; et al.	Pineal Parenchymal Tumors - Clinical, Pathological, And Therapeutic Aspects	Cancer	142	4.7
**2003**	Jouvet, A; et al.	Papillary Tumor Of The Pineal Region	American Journal Of Surgical Pathology	137	6.9
**2000**	Jouvet, A; et al.	Pineal Parenchymal Tumors: A Correlation Of Histological Features With Prognosis In 66 Cases	Brain Pathology	128	5.6
**2000**	Fauchon, F; et al.	Parenchymal Pineal Tumors: A Clinicopathological Study Of 76 Cases	International Journal Of Radiation Oncology∗ Biology∗ Physics	125	5.4
**2006**	Fevre-Montange, M; et al.	Prognosis And Histopathologic Features In Papillary Tumors Of The Pineal Region: A Retrospective Multicenter Study Of 31 Cases	Journal Of Neuropathology & Experimental Neurology	109	6.4
**1980**	Borit, A; et al.	The Separation Of Pineocytoma From Pineoblastoma	Cancer	99	2.3
**2002**	Lutterbach, J; et al.	Malignant Pineal Parenchymal Tumors In Adult Patients: Patterns Of Care And Prognostic Factors	Neurosurgery	86	4.1
**2006**	Fevre-Montange, M; et al.	Microarray Analysis Reveals Differential Gene Expression Patterns In Tumors Of The Pineal Region	Journal Of Neuropathology & Experimental Neurology	76	4.5
**2006**	Hasselblatt, M; et al.	Immunohistochemical Profile And Chromosomal Imbalances In Papillary Tumors Of The Pineal Region	Neuropathology And Applied Neurobiology	73	4.3
**1995**	Mena, H; et al.	Tumors Of Pineal Parenchymal-Cells - A Correlation Of Histological Features, Including Nucleolar Organizer Regions, With Survival In 35 Cases	Human Pathology	69	2.5
**2002**	Hasegawa, T; et al.	The Role Of Radiosurgery For The Treatment Of Pineal Parenchymal Tumors	Neurosurgery	66	3.1
**1994**	Jouvet, A; et al.	Structural And Ultrastructural Characteristics Of Human Pineal-Gland, And Pineal Parenchymal Tumors	Acta Neuropathologica	64	2.2
**2004**	Shibahara, J; et al.	Papillary Neuroepithelial Tumor Of The Pineal Region. A Case Report	Acta Neuropathologica	61	3.2
**1986**	Perentes, E; et al.	S-Antigen Immunoreactivity In Human Pineal Glands And Pineal Parenchymal Tumors - A Monoclonal-Antibody Study	Acta Neuropathologica	58	1.6
**2008**	Chang, Ah; et al.	Mr Imaging Of Papillary Tumor Of The Pineal Region	American Journal Of Neuroradiology	55	3.7
**2007**	Roncaroli, F; et al.	Papillary Tumor Of The Pineal Region And Spindle Cell Oncocytoma Of The Pituitary: New Tumor Entities In The (2007) Who Classification	Brain Pathology	53	3.3
**2012**	Tate, M; et al.	The Long-Term Postsurgical Prognosis Of Patients With Pineoblastoma	Cancer	44	4
**2007**	Dagnew, E; et al.	Papillary Tumors Of The Pineal Region: Case Report	Neurosurgery	43	2.7
**2001**	Rickert, Ch; et al.	Comparative Genomic Hybridization In Pineal Parenchymal Tumors	Genes Chromosomes & Cancer	42	1.9
**2014**	De Kock, L; et al.	Germ-Line And Somatic Dicer1 Mutations In Pineoblastoma	Acta Neuropathologica	41	4.6
**2008**	Boco, T; et al.	Papillary Tumor Of The Pineal Region	Neuropathology	41	2.7
**2012**	Sabbaghian, N; et al.	Germline Dicer1 Mutation And Associated Loss Of Heterozygosity In A Pineoblastoma	Journal Of Medical Genetics	40	3.6
**2008**	Fakhran, S; et al.	Pineocytoma Mimicking A Pineal Cyst On Imaging: True Diagnostic Dilemma Or A Case Of Incomplete Imaging?	American Journal Of Neuroradiology	40	2.7
**2006**	Reyns, N; et al.	The Role Of Gamma Knife Radiosurgery In The Treatment Of Pineal Parenchymal Tumors	Acta Neurochirurgica	40	2.4
**2008**	Gilheeney, Sw; et al.	Outcome Of Pediatric Pineoblastoma After Surgery, Radiation And Chemotherapy	Journal Of Neuro-Oncology	39	2.6
**2006**	Kuchelmeister, K; et al.	Papillary Tumour Of The Pineal Region: Histodiagnostic Considerations	Neuropathology And Applied Neurobiology	39	2.3
**2009**	Sato, Ts; et al.	Papillary Tumor Of The Pineal Region: Report Of A Rapidly Progressive Tumor With Possible Multicentric Origin	Pediatric Radiology	38	2.7
**2004**	Deshmukh, Vr; et al.	Diagnosis And Management Of Pineocytomas	Neurosurgery	38	2
**1982**	Trojanowski, Jq; et al.	Malignant Pineocytoma With Prominent Papillary Features	Cancer	38	0.9
**1986**	Korf, Hw; et al.	S-Antigen-Like Immunoreactivity In A Human Pineocytoma	Acta Neuropathologica	37	1
**1975**	Nielsen, Sl; et al.	Ultrastructure Of A Pineocytoma	Journal Of Neuropathology & Experimental Neurology	36	0.8
**2020**	Li, Bk; et al.	Pineoblastoma Segregates Into Molecular Sub-Groups With Distinct Clinico-Pathologic Features: A Rare Brain Tumor Consortium Registry Study	Acta Neuropathologica	35	12
**1990**	Vaquero, J; et al.	Papillary Pineocytoma	Journal Of Neurosurgery	35	1.1
**2008**	Inoue, T; et al.	Papillary Tumor Of The Pineal Region: A Case Report	Brain Tumor Pathology	34	2.3
**2008**	Buffenoir, K; et al.	Papillary Tumor Of The Pineal Region In A Child: Case Report And Review Of The Literature	Child'S Nervous System	34	2.3
**1979**	Kline, Kt; et al.	Pineoblastoma - Electron-Microscopic Study	Cancer	34	0.8
**2013**	Fauchon, F; et al.	Role Of Surgery, Radiotherapy And Chemotherapy In Papillary Tumors Of The Pineal Region: A Multicenter Study	Journal Of Neuro-Oncology	33	3.3
**2010**	Dahiya, S; et al.	Pineal Tumors	Advances In Anatomic Pathology	33	2.5
**2007**	Hinkes, Bg; et al.	Childhood Pineoblastoma: Experiences From The Prospective Multicenter Trials Hit-Skk87, Hit-Skk92 And Hit91	Journal Of Neuro-Oncology	33	2.1
**1995**	Chiechi, Mv; et al.	Pineal Parenchymal Tumors - Ct And Mr Features	Journal Of Computer Assisted Tomography	33	1.2
**1970**	Rubinstein, Lj; et al.	Gangliogliomatous Differentiation In A Pineocytoma	The Journal Of Pathology	33	0.6
**2014**	Goschzik, T; et al.	Pten Mutations And Activation Of The Pi3K/Akt/Mtor Signaling Pathway In Papillary Tumors Of The Pineal Region	Journal Of Neuropathology & Experimental Neurology	31	3.4
**2012**	Rodjan, F; et al.	Trilateral Retinoblastoma: Neuroimaging Characteristics And Value Of Routine Brain Screening On Admission	Journal Of Neuro-Oncology	31	2.8
**2007**	Kawahara, I; et al.	Papillary Tumor Of The Pineal Region - Case Report	Neurologia Medico-Chirurgica	30	1.9
**2005**	Lee, Jyk; et al.	Management And Survival Of Pineoblastoma: An Analysis Of 34 Adults From The Brain Tumor Registry Of Japan	Neurologia Medico-Chirurgica	30	1.7
**1994**	Numoto, Rt	Pineal Parenchymal Tumors - Cell-Differentiation And Prognosis	Journal Of Cancer Research And Clinical Oncology	30	1
**2018**	Snuderl, M; et al.	Recurrent Homozygous Deletion Of Drosha And Microduplication Of Pde4Dip In Pineoblastoma	Nature Communications	29	5.8
**2010**	Stoiber, Em; et al.	Long Term Outcome Of Adolescent And Adult Patients With Pineal Parenchymal Tumors Treated With Fractionated Radiotherapy Between 1982 And 2003 - A Single Institution'S Experience	Radiation Oncology	29	2.2
**2000**	Nakamura, M; et al.	Neuroradiological Characteristics Of Pineocytoma And Pineoblastoma	Neuroradiology	29	1.3
**1996**	Amoaku, Wmk; et al.	Trilateral Retinoblastoma - A Report Of Five Patients	Cancer	29	1.1
**2016**	Mallick, S; et al.	Patterns Of Care And Survival Outcomes In Patients With Pineal Parenchymal Tumor Of Intermediate Differentiation: An Individual Patient Data Analysis	Radiotherapy And Oncology	28	4
**2011**	Poulgrain, K; et al.	Papillary Tumour Of The Pineal Region	Journal Of Clinical Neuroscience	28	2.3
**2019**	Lee, Jc; et al.	Recurrent Kbtbd4 Small In-Frame Insertions And Absence Of Drosha Deletion Or Dicer1 Mutation Differentiate Pineal Parenchymal Tumor Of Intermediate Differentiation (Pptid) From Pineoblastoma	Acta Neuropathologica	27	6.8
**2017**	Raleigh, Dr; et al.	Histopathologic Review Of Pineal Parenchymal Tumors Identifies Novel Morphologic Subtypes And Prognostic Factors For Outcome	Neuro-Oncology	27	4.5
**2009**	Sharma, Mc; et al.	Papillary Tumor Of The Pineal Region - A Recently Described Entity: A Report Of Three Cases And Review Of The Literature	Clinical Neuropathology	27	1.9
**2011**	Komakula, S; et al.	Pineal Parenchymal Tumor Of Intermediate Differentiation: Imaging Spectrum Of An Unusual Tumor In 11 Cases	Neuroradiology	26	2.2
**2009**	Cerase, A; et al.	Neuroradiological Follow-Up Of The Growth Of Papillary Tumor Of The Pineal Region: A Case Report	Journal Of Neuro-Oncology	26	1.9
**2020**	Pfaff, E; et al.	Molecular Subgrouping Of Primary Pineal Parenchymal Tumors Reveals Distinct Subtypes Correlated With Clinical Parameters And Genetic Alterations	Acta Neuropathologica	25	8.3
**2020**	Thomas, C; et al.	Desmoplastic Myxoid Tumor, Smarcb1-Mutant: Clinical, Histopathological And Molecular Characterization Of A Pineal Region Tumor Encountered In Adolescents And Adults	Acta Neuropathologica	25	8.3
**2016**	Heim, S; et al.	Papillary Tumor Of The Pineal Region: A Distinct Molecular Entity	Brain Pathology	25	3.6
**2012**	Fevre-Montange, M; et al.	Utility Of Ki67 Immunostaining In The Grading Of Pineal Parenchymal Tumors: A Multicentre Study	Neuropathology And Applied Neurobiology	25	2.3
**2017**	Parikh, Ka; et al.	Pineoblastoma-The Experience At St. Jude Children'S Research Hospital	Neurosurgery	24	4
**2017**	Mynarek, M; et al.	Evaluation Of Age-Dependent Treatment Strategies For Children And Young Adults With Pineoblastoma: Analysis Of Pooled European Society For Paediatric Oncology (Siop-E) And Us Head Start Data	Neuro-Oncology	24	4
**2010**	Clark, Aj; et al.	Tumor Control After Surgery And Radiotherapy For Pineocytoma	Journal Of Neurosurgery	24	1.8
**2008**	Fevre-Montange, M; et al.	Pineocytoma And Pineal Parenchymal Tumors Of Intermediate Differentiation Presenting Cytologic Pleomorphism: A Multicenter Study	Brain Pathology	24	1.6
**2006**	Pusztaszeri, M; et al.	Pineal Parenchymal Tumors Of Intermediate Differentiation In Adults: Case Report And Literature Review	Neuropathology	24	1.4
**1989**	Sreekantaiah, C; et al.	Interstitial Deletion Of Chromosome-11Q In A Pineoblastoma	Cancer Genetics And Cytogenetics	24	0.7
**1985**	Whittle, Ir; et al.	Concurrent Pineoblastoma And Unilateral Retinoblastoma - A Forme Fruste Of Trilateral Retinoblastoma	Neurosurgery	24	0.6
**2008**	Santarius, T; et al.	Papillary Tumour Of The Pineal Region	British Journal Of Neurosurgery	23	1.5
**2002**	Yamane, Y; et al.	Immunohistochemical Characterization Of Pineal Parenchymal Tumors Using Novel Monoclonal Antibodies To The Pineal Body	Neuropathology	23	1.1
**1994**	Kuchelmeister, K; et al.	Pleomorphic Pineocytoma With Extensive Neuronal Differentiation - Report Of 2 Cases	Acta Neuropathologica	23	0.8
**2020**	Liu, Apy; et al.	Risk-Adapted Therapy And Biological Heterogeneity In Pineoblastoma: Integrated Clinico-Pathological Analysis From The Prospective, Multi-Center Sjmb03 And Sjyc07 Trials	Acta Neuropathologica	22	7.3
**2010**	Clark, Aj; et al.	Factors Influencing Overall Survival Rates For Patients With Pineocytoma	Journal Of Neuro-Oncology	22	1.7
**2009**	Yano, H; et al.	Clinicopathological Features From Long-Term Observation Of A Papillary Tumor Of The Pineal Region (Ptpr): A Case Report	Brain Tumor Pathology	22	1.6
**1998**	Kees, Ur; et al.	A New Pineoblastoma Cell Line, Per-480, With Der(10)T(10; 17), Der(16)T(1; 16), And Enhanced Myc Expression In The Absence Of Gene Amplification	Cancer Genetics And Cytogenetics	22	0.9
**1994**	Min, Kw; et al.	Pineal Parenchymal Tumors - An Ultrastructural-Study With Prognostic Implications	Ultrastructural Pathology	22	0.8
**1981**	Sobel, Ra; et al.	Pineoblastoma With Ganglionic And Glial Differentiation - Report Of 2 Cases	Acta Neuropathologica	22	0.5
**1979**	Borit, A; et al.	Pineocytoma With Astrocytomatous Differentiation	Journal Of Neuropathology & Experimental Neurology	22	0.5
**2014**	Farnia, B; et al.	Clinical Outcomes And Patterns Of Failure In Pineoblastoma: A 30-Year, Single-Institution Retrospective Review	World Neurosurgery	21	2.3
**2014**	Ito, T; et al.	Clinicopathologic Study Of Pineal Parenchymal Tumors Of Intermediate Differentiation	World Neurosurgery	21	2.3
**2014**	Heim, S; et al.	Increased Mitotic And Proliferative Activity Are Associated With Worse Prognosis In Papillary Tumors Of The Pineal Region	American Journal Of Surgical Pathology	21	2.3
**2009**	Kim, Bs; et al.	Pineal Parenchymal Tumor Of Intermediate Differentiation Showing Malignant Progression At Relapse	Neuropathology	21	1.5
**2009**	Nakamura, H; et al.	Successful Treatment Of Neoadjuvant Therapy For Papillary Tumor Of The Pineal Region	Brain Tumor Pathology	21	1.5
**1998**	Kurisaka, M; et al.	Combination Chemotherapy (Cisplatin, Vinblastin) And Low-Dose Irradiation In The Treatment Of Pineal Parenchymal Cell Tumors	Child'S Nervous System	21	0.8
**1998**	Fevre-Montange, M; et al.	Immunohistochemical, Ultrastructural, Biochemical And In Vitro Studies Of A Pineocytoma	Acta Neuropathologica	21	0.8
**2021**	Liu, Apy; et al.	Clinical And Molecular Heterogeneity Of Pineal Parenchymal Tumors: A Consensus Study	Acta Neuropathologica	20	10
**2014**	Watanabe, T; et al.	Pineal Parenchymal Tumor Of Intermediate Differentiation: Treatment Outcomes Of Five Cases	Molecular And Clinical Oncology	20	2.2
**2008**	Arivazhagan, A; et al.	Pineal Parenchymal Tumors - Utility Of Immunohistochemical Markers In Prognostication	Clinical Neuropathology	20	1.3
**1974**	Banerjee, Ak; et al.	Pineoblastoma With Spontaneous Intra And Extracranial Metastasis	Journal Of Pathology	20	0.4
**2016**	Yu, T; et al.	Twenty-Seven Cases Of Pineal Parenchymal Tumors Of Intermediate Differentiation: Mitotic Count, Ki-67 Labelling Index And Extent Of Resection Predict Prognosis	Journal Of Neurology Neurosurgery And Psychiatry	19	2.7
**2011**	Gutenberg, A; et al.	Common Molecular Cytogenetic Pathway In Papillary Tumors Of The Pineal Region (Ptpr)	Brain Pathology	19	1.6
**2011**	Rickard, Ka; et al.	Papillary Tumor Of The Pineal Region: Two Case Studies And A Review Of The Literature	Annals Of Clinical & Laboratory Science	19	1.6
**2010**	Fukuda, T; et al.	Expression Of Hydroxyindole-O-Methyltransferase Enzyme In The Human Central Nervous System And In Pineal Parenchymal Cell Tumors	Journal Of Neuropathology & Experimental Neurology	19	1.5
**2008**	Senft, C; et al.	Pineal Parenchymal Tumor Of Intermediate Differentiation: Diagnostic Pitfalls And Discussion Of Treatment Options Of A Rare Tumor Entity	Neurosurgical Review	19	1.3
**2006**	Anan, M; et al.	Postoperative Adjuvant Treatment For Pineal Parenchymal Tumour Of Intermediate Differentiation	Journal Of Clinical Neuroscience	19	1.1
**1992**	Rainho, Ca; et al.	Cytogenetic Study Of A Pineocytoma	Cancer Genetics And Cytogenetics	19	0.6
**2012**	Montange, Mf; et al.	Histopathologic And Ultrastructural Features And Claudin Expression In Papillary Tumors Of The Pineal Region: A Multicenter Analysis	American Journal Of Surgical Pathology	17	1.5
**2011**	Han, Sj; et al.	Pathology Of Pineal Parenchymal Tumors	Neurosurgery Clinics Of North America	17	1.4
**2011**	Tate, Mc; et al.	Contemporary Management Of Pineoblastoma	Neurosurgery Clinics Of North America	17	1.4

CC: Citation Count, C.Y: Citation per Year.

### 3.2 Top 10 most cited articles

The top 10 most cited articles were published in 9 different journals, the oldest and most cited was published in 1979, with the most recent one published in 2006. Two authors each first-authored two of the top 10 most cited articles, Anne Jouvet from Hôpital Neurologique, France, and Michelle Fèvre-Montange from INSERM, France (Table 2).

**Table 2:** Top 10 cited articles of Pineal Parenchymal Tumors.

#.	Year	Authors	Article Title	Journal	CC	C.Y
**1.**	1979	Herrick, Mk; et al.	Cytological Differentiating Potential Of Pineal Parenchymal Neoplasms (True Pinealomas) - Clinico-Pathological Study Of 28 Tumors	Brain: A Journal Of Neurology	152	3.5
**2.**	1993	Schild, Se; et al.	Pineal Parenchymal Tumors - Clinical, Pathological, And Therapeutic Aspects	Cancer	142	4.7
**3.**	2003	Jouvet, A; et al.	Papillary Tumor Of The Pineal Region	American Journal Of Surgical Pathology	137	6.9
**4.**	2000	Jouvet, A; et al.	Pineal Parenchymal Tumors: A Correlation Of Histological Features With Prognosis In 66 Cases	Brain Pathology	128	5.6
**5.**	2000	Fauchon, F; et al.	Parenchymal Pineal Tumors: A Clinicopathological Study Of 76 Cases	International Journal Of Radiation Oncology∗ Biology∗ Physics	125	5.4
**6.**	2006	Fevre-Montange, M; et al.	Prognosis And Histopathologic Features In Papillary Tumors Of The Pineal Region: A Retrospective Multicenter Study Of 31 Cases	Journal Of Neuropathology & Experimental Neurology	109	6.4
**7.**	1980	Borit, A; et al.	The Separation Of Pineocytoma From Pineoblastoma	Cancer	99	2.3
**8.**	2002	Lutterbach, J; et al.	Malignant Pineal Parenchymal Tumors In Adult Patients: Patterns Of Care And Prognostic Factors	Neurosurgery	86	4.1
**9.**	2006	Fevre-Montange, M; et al.	Microarray Analysis Reveals Differential Gene Expression Patterns In Tumors Of The Pineal Region	Journal Of Neuropathology & Experimental Neurology	76	4.5
**10.**	2006	Hasselblatt, M; et al.	Immunohistochemical Profile And Chromosomal Imbalances In Papillary Tumors Of The Pineal Region	Neuropathology And Applied Neurobiology	73	4.3

### 3.3 Highest 10 citation-per-year articles

The 10 highest citation per year (C.Y) articles were published in 5 journals, by 8 institutions, 8 unique first authors, and from range of years from 2000 to 2021. The highest contributing journal was Acta Neuropathologica with 6 articles. The article with the highest C.Y (12 citations/year) was published in 2020 by Li et al. from The Hospital for Sick Children, University of Toronto titled " Pineoblastoma Segregates into Molecular Sub-Groups with Distinct Clinico-Pathologic Features: A Rare Brain Tumor Consortium Registry Study” (Table 3).

**Table 3:** The 10 highest articles in Citation per Year.

Year	Authors	Title	Journal	Institution	C.Y	CC
**2020**	Li, Bk; et al.	Pineoblastoma Segregates into Molecular Sub-Groups with Distinct Clinico-Pathologic Features: A Rare Brain Tumor Consortium Registry Study	Acta Neuropathologica	University of Toronto	12	35
**2021**	Liu, Apy; et al.	Clinical And Molecular Heterogeneity of Pineal Parenchymal Tumors: A Consensus Study	Acta Neuropathologica	St. Jude Children's Research Hospital	10	20
**2020**	Pfaff, E; et al.	Molecular Subgrouping of Primary Pineal Parenchymal Tumors Reveals Distinct Subtypes Correlated With Clinical Parameters And Genetic Alterations	Acta Neuropathologica	Heidelberg University Hospital	8.3	25
**2020**	Thomas, C; et al.	Desmoplastic Myxoid Tumor, Smarcb1-Mutant: Clinical, Histopathological and Molecular Characterization Of A Pineal Region Tumor Encountered In Adolescents And Adults	Acta Neuropathologica	University Hospital Münster	8.3	25
**2020**	Liu, Apy; et al.	Risk-Adapted Therapy and Biological Heterogeneity In Pineoblastoma: Integrated Clinico-Pathological Analysis From The Prospective, Multi-Center Sjmb03 And Sjyc07 Trials	Acta Neuropathologica	St. Jude Children's Research Hospital	7.3	22
**2003**	Jouvet, A; et al.	Papillary Tumor of The Pineal Region	American Journal of Surgical Pathology	Hôpital Neurologique	6.9	137
**2019**	Lee, Jc; et al.	Recurrent Kbtbd4 Small In-Frame Insertions and Absence Of Drosha Deletion Or Dicer1 Mutation Differentiate Pineal Parenchymal Tumor Of Intermediate Differentiation (Pptid) From Pineoblastoma	Acta Neuropathologica	University of California	6.8	27
**2006**	Fevre-Montange, M; et al.	Prognosis And Histopathologic Features In Papillary Tumors Of The Pineal Region: A Retrospective Multicenter Study Of 31 Cases	Journal Of Neuropathology & Experimental Neurology	INSERM	6.4	109
**2018**	Snuderl, M; et al.	Recurrent Homozygous Deletion of Drosha And Microduplication Of Pde4Dip In Pineoblastoma	Nature Communications	NYU Langone Health	5.8	29
**2000**	Jouvet, A; et al.	Pineal Parenchymal Tumors: A Correlation of Histological Features With Prognosis In 66 Cases	Brain Pathology	Hôpital Neurologique	5.6	128

### 3.4 Journals

**Fig. 2:**
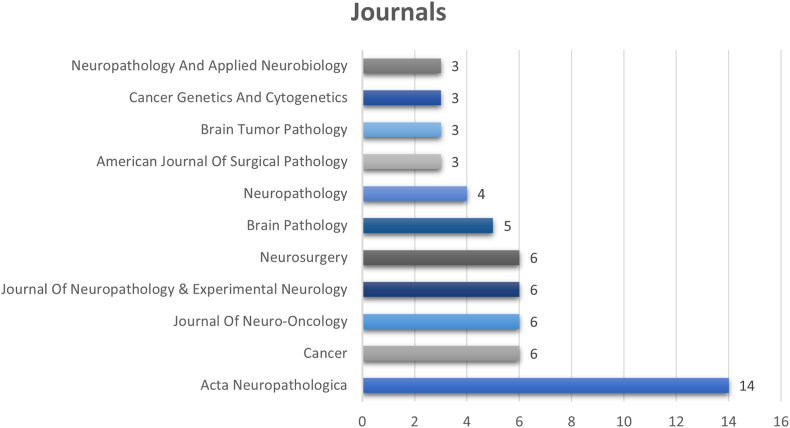
Horizontal Bar Chart Depicting Journals with Three or more Publications.

The number of journals that contributed to the top 100 most cited articles is 42 unique journals. The journal with the highest number of publications was Acta Neuropathologica, with 14 articles, followed by Cancer, Journal of Neuro-Oncology, Journal of Neuropathology & Experimental Neurology, and Neurosurgery, each with 6 publications (Fig. 2).

### 3.5 Countries

**Fig. 3:**
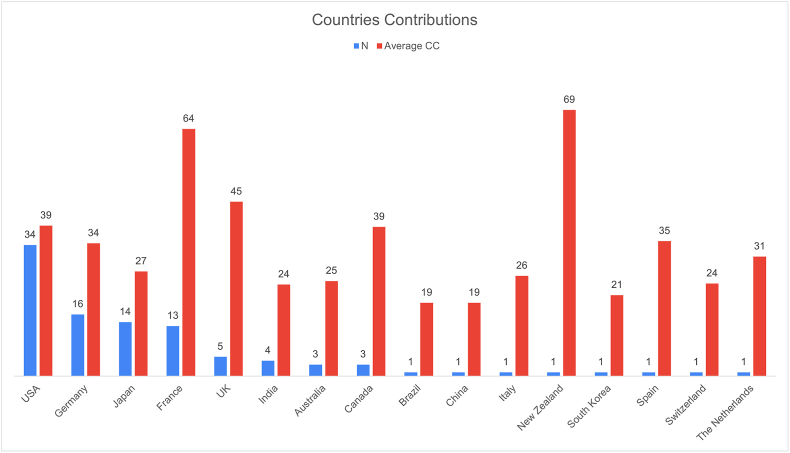
Bar chart illustrating citation metrics by country among the top 100 most cited articles.

Sixteen countries published the top 100 most cited articles, with eight countries contributing three or more articles. The country with the most published articles is the USA, with 34 articles, followed by Germany with 16 articles. In terms of citation count per country, the USA has the highest total number of citations and C.Y metric out of all articles, with 1326 citations and 96 C.Y, respectively. However, in average C.Y, Canada appears first, with 6.7 citations per year per published article (Table 4). In average citation count per article per country, New Zealand ranks first with 69 citations (1 article), followed by France, with 64 citations (13 articles) (Fig. 3).

**Table 4:** Countries total citation count.

Country	CC	C.Y	Average C.Y
USA	1326	96	2.8
France	833	45	3.5
Germany	551	52	3.2
Japan	380	24	1.7
UK	226	8.7	1.7
Canada	116	20	6.7
India	95	7.6	1.9
Australia	74	4	1.3
New Zealand	69	2.5	2.5
Spain	35	1.1	1.1
The Netherlands	31	2.8	2.8
Italy	26	1.9	1.9
South Korea	21	1.5	1.5
Switzerland	24	1.4	1.4
Brazil	19	0.6	0.6
China	19	2.7	2.7

### 3.6 Institutions

**Fig. 4:**
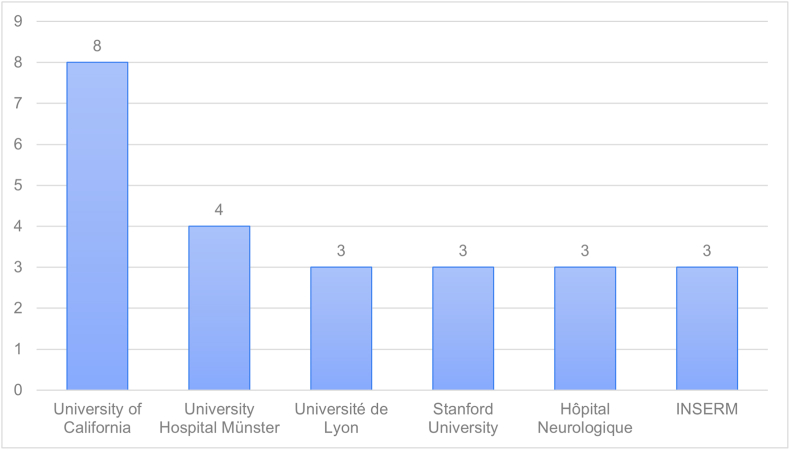
– Bar chart depicting top contributing first-author affiliated institutions among the 100 most cited articles.

Seventy-four institutions were the first-author affiliated institutions in the top 100 most cited articles. University of California was the institution with the most contributions, 8 articles, followed by University Hospital Münster, with 4 articles (Fig. 4).

### 3.7 Years of publication

**Fig. 5:**
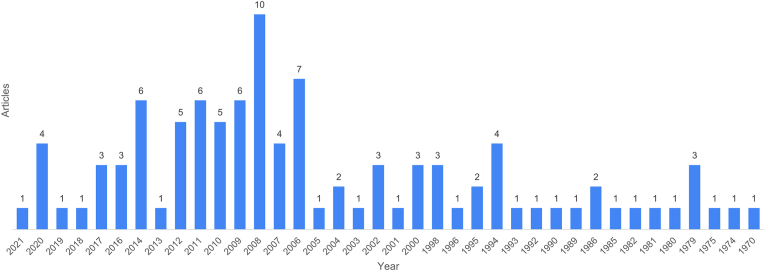
– Bar chart illustrating years of publication (1970–2021).

The included articles were published in the years 1970–2021. The most prolific year was 2008, which had 10 publications, followed by 2006, which had 7 publications. The 2000–2010 decade produced the greatest number of influential publications (Fig. 5).

### 3.8 Categories

**Fig. 6:**
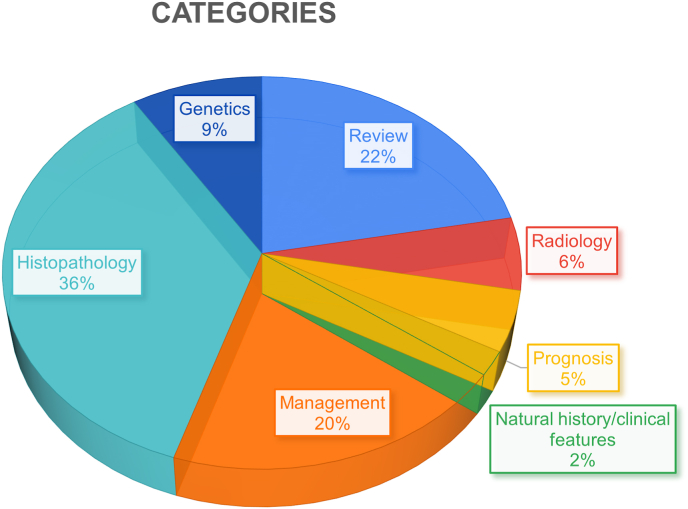
Pie chart depicting categorical distribution of articles.

Categorical distribution of the included articles revealed a predominant focus of the literature on histopathology, accounting for 36 % of the articles. The least studied category was natural history/clinical features (Fig. 6). Regarding the influence of categories, mean, total citation count, and total C.Y received per article is highest in histopathology articles, with 45, 1618, and 109 citations, respectively. For mean C.Y, Genetics category ranks higher slightly above histopathology, with 3.1 mean C.Y (Table 5).

**Table 5:** Impact of category.

Category	N	Mean CC	Total CC	Mean C.Y	Total C.Y
**Genetics**	**9**	**31**	**275**	**3.1**	**28**
**Histopathology**	**36**	**45**	**1618**	**3.0**	**109**
**Management**	**20**	**35**	**706**	**2.5**	**50**
**Natural history/clinical features**	**2**	**34**	**67**	**1.7**	**3**
**Prognosis**	**5**	**34**	**171**	**2.76**	**14**
**Radiology**	**6**	**35**	**209**	**2.2**	**13**
**Review**	**22**	**36**	**799**	**2.4**	**53**

### 3.9 Types of articles

**Fig. 7:**
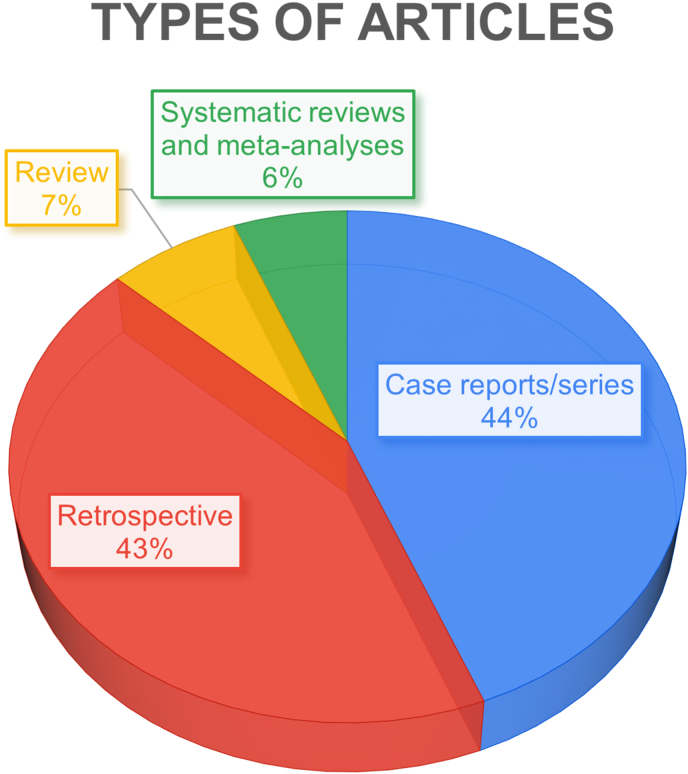
Pie chart illustrating types of articles included.

Case reports/series were the dominant type of articles included in the bibliometric analysis. It is followed by retrospective studies with a difference of 1 % only (Fig. 7). In impact, the type of articles that received the highest average citation count is retrospective studies, with 45 CC per article. The least was systematic reviews and meta-analyses with 25 mean CC. However, given that all systematic reviews included in this study were published after 2007, they exceed all other types of articles in average C.Y metric, with 3.9 citations (Table 6).

**Table 6:** Impact of article type.

Type	N	Mean CC	Total CC	Mean C.Y	Total C.Y
**Case Report/Series**	**44**	**35**	**1544**	**1.7**	**76**
**Retrospective**	**43**	**45**	**1921**	**3.5**	**151**
**Review**	**7**	**32.7**	**229**	**3.0**	**32**
**Systematic Review/Meta-Analyses**	**6**	**25**	**151**	**3.9**	**24**

### 3.10 Pathology

**Fig. 8:**
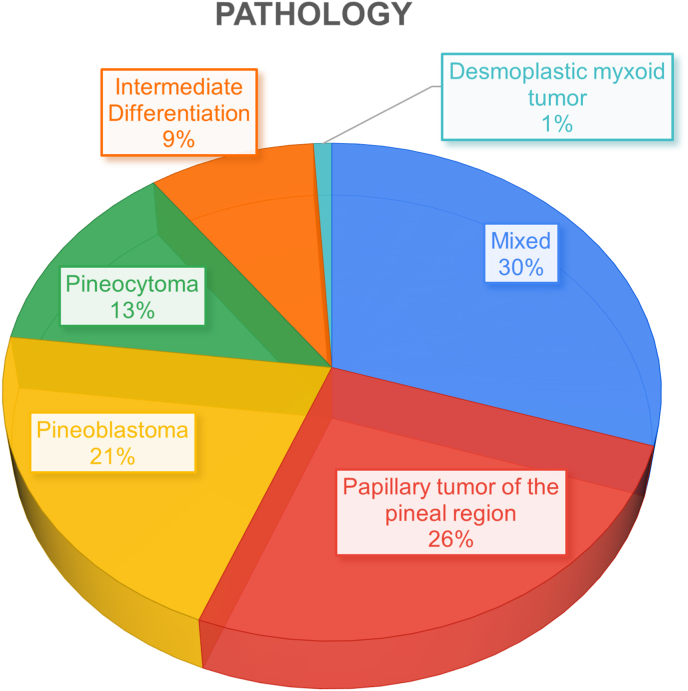
– Pie chart depicting pathology distribution of articles.

The most frequent single pathology predominantly discussed in the included articles was papillary tumor of the pineal region, whereas the greatest number of articles, 30 %, discussed a mix of 2 or more pathologies in the same paper. The least single pathology studied was desmoplastic myxoid tumors, with only 1 article (Fig. 8). For impact, the highest influence was in articles discussing mixed pineal pathologies without having a specific focus on one pathology. They received the highest mean, total citation count, and total C.Y, with 52, 1571, and 92 citations, respectively. There was only 1 article which discussed desmoplastic myxoid tumor which received the highest average C.Y, with 8.3 citations (Table 7).

**Table 7:** Impact of pathology.

Pathology	N	Mean CC	Total CC	Mean C.Y	Total C.Y
**Mixed**	**30**	**52**	**1571**	**3.1**	**92**
**Papillary Tumor of the Pineal Region**	**26**	**41**	**1059**	**2.8**	**73**
**Pineoblastoma**	**21**	**29**	**605**	**3.0**	**63**
**Pineocytoma**	**13**	**30**	**388**	**1.2**	**15**
**Pineal Parenchymal Tumors of Intermediate Differentiation**	**9**	**22**	**197**	**2.1**	**19**
**Desmoplastic myxoid tumor**	**1**	**25**	**25**	**8.3**	**8.3**

### 3.11 Countries and interests

**Fig. 9:**
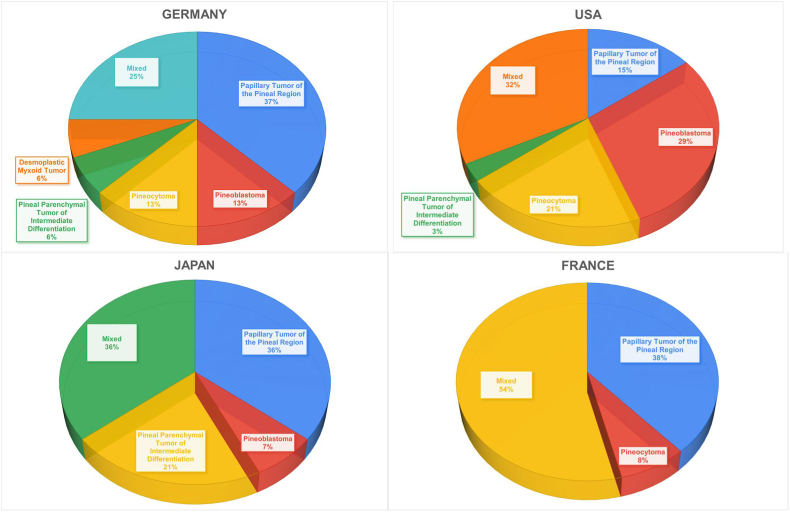
– Pie chart showing countries interest.

When analyzed, the countries that contributed more than 10 articles show patterns of interest or focus on specific pathologies. USA, Germany, Japan, and France contributed 34, 16, 14, 13 articles, respectively. Germany is the only country which had predominantly pathology-specific articles, focusing 37 % of its literature on papillary tumor of the pineal region, and is the only country which contributed to desmoplastic myxoid tumor, with the one the only article included in this bibliometric analysis (Fig. 9).

## 4 Discussion

This article offers an analysis of the top 100 most cited articles on pineal parenchymal tumors, as indexed in the WOS database. It delineates research hotspots and identifies areas of deficiency. The insights derived from this analysis are instrumental in guiding targeted research efforts, thereby enabling future researchers to significantly enhance the body of literature in this field and achieve high-impact contributions.

The current body of literature in neurosurgery, as revealed through bibliometric analyses, demonstrates a marked scarcity of high-impact research specifically addressing pineal parenchymal tumors. Research output in this area is significantly lower compared to other neurosurgical topics. For example, a comparative analysis of articles indexed in databases such as WOS and Scopus shows a clear disparity: while search results for a variety of neurosurgical subjects range from 1733 to 49,799 articles, those on pineal parenchymal tumors yield only 573. Moreover, these articles exhibit a lower citation impact, with an average of 38.45 citations, compared to a broader citation range of 66.23 to 1485.94 (Table 8). Citation dynamics differ from more common neurosurgical entities such as hydrocephalus, where larger patient volumes and surgical studies naturally accrue higher citations. The predominance of case reports and series (44 %) (Fig. 6) within the existing literature further underscores the rarity of these tumors and highlights the lack of large-scale studies and clinical trials, limiting the development of evidence-based treatment guidelines and prognostic models. This significant gap in research underscores the urgent need for more rigorous investigations and resource allocation to better understand and improve outcomes for patients with pineal parenchymal tumors (Hamzah et al., 2023; Hirano et al., 2024; Alnefaie et al., 2022; Albrahim et al., 2022; Akmal et al., 2020; Abdullah et al., 2024).

**Table 8:** Comparison of pineal parenchymal tumor articles with other neurosurgical bibliometric studies.

Article	Topic	Search outcome (No. Articles)	Average Citation Count
**This article**	Pineal Parenchymal Tumors	573	38.45
**Yudai Hirano et al. 2023** (Hirano et al., 2024)	Intracranial artery stenosis and intracranial atherosclerosis	49,799	213.84
**Nada**Alnefaie et al., 2022 (Alnefaie et al., 2022)	Neurofibromatosis	12,718	289.57
**Mohammed**Albrahim et al., 2022 (Albrahim et al., 2022)	Hydrocephalus	12,044	201.77
**Manahil**Akmal et al., 2020 (Akmal et al., 2020)	Glioblastome multiforme	Not reported	1485.94
**Abdulaziz**Hamzah et al., 2023 (Hamzah et al., 2023)	Hemangioblastoma of the central nervous system	4023	87.81
**Abdullah Abdullah et al. 2024** (Abdullah et al., 2024)	Carotid body tumors	1733	66.23

The United States emerged as the leading contributor to pineal parenchymal tumor research, a trend mirrored in other neurosurgical bibliometric analyses. For instance, a bibliometric analysis of the top 100 most cited articles on hydrocephalus highlighted that the United States accounted for over half of the contributions, followed by Canada and European countries like Germany and the UK. (Albrahim et al., 2022) This dominance is consistent with other analyses, such as those on hemangioblastomas of the central nervous system, where the USA again had the highest publication output (Hamzah et al., 2023). While the contribution of the US is invaluable, it is important for other countries to focus on publishing high-quality articles that represent their specific data. This would enhance diversity in the research and help uncover potential disparities or heterogeneity in pineal parenchymal tumors. Such international efforts could drive significant advancements in the diagnostic and interventional aspects of these rare tumors.

Beyond geographical trends, the thematic focus of influential studies also offers valuable insights. In the bibliometric analyses reviewed, the most studied categories varied depending on the research area: surgical management was predominant in hydrocephalus studies (Albrahim et al., 2022), whereas histopathological research dominated both hemangioblastoma of the central nervous system (Hamzah et al., 2023) and pineal parenchymal tumors, as revealed in our analysis. The predominance of histopathology articles, accounting for 36 % of the research, alongside the lower number of articles and citation counts for studies on management and natural history/clinical features, reflects a focus on basic science and tumor classification rather than interventional research. This trend is likely driven by the inherent challenges in studying rare tumors, such as difficulties in patient recruitment and the need for standardized treatment protocols. Accordingly, there is a critical need for future high-quality research focused on clinical presentation, surgical challenges, and patient outcomes—areas that remain significantly underexplored despite their crucial relevance for improving diagnosis and treatment. Furthermore, the high citation rates for studies addressing mixed pathologies emphasize their importance, as these studies involve comprehensive morphological and immunohistochemical analyses, highlighting tumor heterogeneity. It is important to recognize that citation frequency does not inherently correlate with clinical utility. While histopathological studies dominate the most-cited list, clinical management studies, though less frequently cited, may exert a more immediate impact on patient care. This highlights the necessity for future high-quality clinical research to complement the existing histological research base.

Recent bibliometric studies in neurosurgery have analyzed publishing patterns in a variety of subspecialties, including journal/author, demographic, and geographic trends, in addition to more general trends. For example, Garg et al. examined articles published in six major neurosurgical journals between 2011 and 2020 and found notable variations in the types of articles, global contributions, and annual publication trends (Garg et al., 2022a, 2022b). Their analyses showed changing publication volumes over time, changing authorship collaborations, and notable international differences in research output. Our study, in contrast, shows comparable patterns of research concentration in a small number of journals and institutions, but it also emphasizes the glaring dearth of clinical and surgical research on pineal parenchymal tumors. These results collectively highlight the need for targeted bibliometric analyses of rare tumors to identify specific gaps and direct future targeted research, even though general neurosurgical publishing patterns are well-characterized. In addition to highlighting particular gaps in clinical management, surgical outcomes, and patient-centered research, our findings on pineal parenchymal tumors are consistent with these more general bibliometric observations. The significance of targeted bibliometric analyses in rare tumor groups is highlighted by this comparison. These analyses serve to supplement extensive neurosurgical evaluations by pinpointing particular shortcomings and directing targeted future research.

There has been a notable shift in focus away from critical areas such as clinical presentation, surgical management and complications—factors that are particularly crucial given the complexity of pineal tumors, which involve intricate anatomical structures and pose significant risks during surgery. The clinical presentation of pineal tumors, vital for accurate diagnosis and management, is underreported in the most-cited articles, highlighting a significant gap in the literature. Instead, most studies concentrate on histopathological classification and molecular analysis, with limited exploration of detailed anatomical relationships, surgical outcomes, and complications. Addressing these gaps is essential for refining surgical techniques and improving patient outcomes.

## 5 Limitations

This study has several limitations inherent to bibliometric analyses. Articles not available online were excluded, which may have led to omission of influential older studies. Additionally, citation counts favor older publications over recent ones, as they have had more time to accumulate citations; this bias was partially mitigated by using the Citations per Year (C.Y) metric. Moreover, citation frequency does not necessarily reflect scientific quality or clinical relevance. Only the Web of Science database was utilized, potentially missing relevant articles indexed elsewhere. Furthermore, obliteration by incorporation—a phenomenon where foundational knowledge becomes so integrated that original articles are no longer explicitly cited—may underestimate the true influence of seminal works. Bibliometric analyses may therefore fail to capture the full impact of foundational discoveries once they become standard components of scientific discourse (Meng et al., 2024)

## 6 Conclusion

This bibliometric analysis provides a comprehensive overview of influential research on pineal parenchymal tumors, highlighting the dominance of histopathological and molecular studies while identifying key contributors and journals. Despite these advances, significant gaps remain in the literature, particularly concerning clinical presentations, surgical treatments, and outcomes, which are under-explored compared to histopathological findings. The heavy reliance on case reports and series underscores the need for more robust and high-quality research to enhance our understanding of these rare tumors. By focusing on surgical challenges, clinical presentation, and patient outcomes, future studies can significantly improve the management and prognosis of pineal parenchymal tumors, ultimately advancing both clinical practice and academic impact in this specialized field.

## Statement of ethics

Institutional Review Board (IRB) approval from King Abdullah International Medical Research Center, Jeddah, Saudi Arabia (KAIMRC-Jeddah) (ID: NRJ23J/191/07) has been obtained.

## Availability of data and materials

All data generated or analyzed during this study are included in this article. Further enquiries can be directed to the corresponding author.

## Funding

This study did not require any funding.

## Declaration of interest

We are pleased to submit our manuscript titled **"**A Bibliometric Analysis of the Top 100 Most Cited Articles on Pineal Parenchymal Tumors Worldwide**"** for consideration in *Clinical Neurology and Neurosurgery.* This manuscript presents a detailed bibliometric analysis of the top 100 most-cited articles on pineal parenchymal tumors, highlighting citation trends, key research areas, and existing gaps in the literature.

Our analysis demonstrates that the majority of research in this field has focused on histopathological classification and molecular studies, while areas such as clinical presentation, surgical approaches, and outcomes remain underrepresented. We believe that this comprehensive review will provide valuable insights for researchers and clinicians alike, encouraging future studies that address these critical gaps to improve patient outcomes in the management of pineal parenchymal tumors.

The manuscript has not been previously published and is not under consideration for publication elsewhere. All authors have read and approved the final version of the manuscript, and there are no conflicts of interest to declare. We have adhered to the submission guidelines for Brain and Spine Journal*,* and we hope that our work will be of interest to the journal's readership.
